# Systematic Overview of *Bacopa monnieri (L.) Wettst.* Dominant Poly-Herbal Formulas in Children and Adolescents

**DOI:** 10.3390/medicines4040086

**Published:** 2017-11-22

**Authors:** James D Kean, Luke A Downey, Con Stough

**Affiliations:** 1 Centre for Human Psychopharmacology, Swinburne University, Melbourne 3122, Australia; ldowney@swin.edu.au (L.A.D.); cstough@swin.edu.au (C.S.); 2 Institute for Breathing and Sleep, Austin Hospital, Melbourne 3084, Australia

**Keywords:** *Bacopa monnieri*, Ayurveda, cognition, behaviour, attention, paediatric, ADHD

## Abstract

**Background:** The Ayurvedic medicinal system employs a holistic approach to health, utilising the synergistic properties of organic resources. Research into the Ayurvedic herb *Bacopa monnieri (L.) Wettst. (B.monnieri)* has reported improvements in cognitive outcomes in child and adult populations. The aim of current review is to systematically assess and critically summarize clinical trials investigating *B.monnieri*-dominant poly-herbal formulas and their effects on the cognition, memory, learning, and behaviour in children and adolescents. **Methods:** Key word searches were performed using PubMed, Scopus, Cochrane Library, DHARA, and CINAHL for publications meeting inclusion criteria up to November 2017. There were no restrictions in study design. Effect sizes were calculated for all significant findings to allow for direct comparisons, and each study was evaluated on design quality. Cognitive and behavioural outcomes were grouped into validated constructs for cross-study comparison. **Results:** Nine trials met inclusion criteria. Five studies reported sufficient data for effect size analysis with most improvements reported in behavioural outcomes. True cognitive abilities and behavioural constructs were reviewed in six studies, with visual perception, impulsivity, and attention demonstrating the greatest improvements. The veracity of the evidence for the formulations reviewed is weakened by inconsistent statistical design and under-reporting of safety and tolerability data (44%). **Conclusions:** The current review extends research supporting *B.monnieri* as a cognitive enhancer and provides modest evidence for the use of *B.monnieri* in poly-herbal preparations for improving cognitive and behavioural outcomes in child and adolescent populations. Greater emphasis on statistical vigour and the reporting of tolerability data are essential for future trials to adequately document poly-herbal treatment efficacy.

## 1. Introduction

Complementary and alternative medicines (CAM) have been widely used throughout history. One common CAM treatment from the *Ayurveda* medicinal system is *Bacopa monnieri (L.) Wettst. (B.monnieri)*, or “*Brahmi*”, from the family *Scrophulariaceae*. *B.monnieri,* is a perennial creeping herb that thrives in damp soils and marshes throughout the subcontinent and is classified as a nootropic (i.e., a cognitive enhancer [1]). The ancient system of medicine, *Ayurveda* (Sanskrit for “science of life”) embraces a holistic approach to healing [2]; a comprehensive treatment system rather than the more commonly accepted single target, single treatment management [3]. In Ayurveda, efficacious CAM treatments are used in combination, rather than individually, to provide a comprehensive synergistic effect [4]. A recent review of research into *B.monnieri* in child and adolescent populations reported its efficacy in improving language behaviour (e.g., vocabulary and verbal comprehension) and memory (visual, meaningful, and overall span) as well as behaviour (hyperactivity and attention) [5]. Furthermore, significant improvements have been noted in adult populations consuming *B.monnieri* in acute [6,7] and chronic [1,8] study designs.

In animal models, *B.monnieri* demonstrated efficacy in treating age-related cognitive decline and dementia [9,10]. These findings led to further research into the cognitive decline of older adults, which demonstrated significant benefits of *B.monnieri* in revitalizing cortical functioning [11]. The mechanism of action purported to be responsible for these cognitive enhancements is linked to the mitigation and protection of neuronal cholinergic systems following administration of *B.monnieri* [9,12,13]. Recent innovative research reported significant improvements in memory in healthy older adults [14], as well as in a population of older adults with age-associated memory impairment [15] following administration with *B.monnieri.* These findings show promise for ageing research, with cholinergic degeneration a major clinical feature of both age-related cognitive decline and Alzheimer’s dementia [16,17].

Poly-herbal research in child and adolescent populations is an area of study requiring increased scrutiny to ensure that the benefits and risks of every vitamin, plant extract, and natural compound have been adequately assessed in stringently controlled clinical trials. This knowledge can then be used to inform the safe treatment of children and adolescents with CAM. The benefits of *B.monnieri* have been well established in terms of distinct cognitive and memory improvements [1,6,7,8,14,18]; with that said, understanding the effects of *B.monnieri* within poly-herbal combination formulas has yet to be considered or reviewed. Additional extracts may provide supplementary health benefits, yet understanding the synergistic effects they provide together is far more difficult to delineate. The aim of the current review was to summarize the findings from clinical trials of poly-herbal formulas with high levels of *B.monnieri* monnieri to strictly assess their effects on the cognition, memory, and behaviour of children and adolescents. Each trial involving *B.monnieri* as a part of a poly-herbal formula was examined in terms of its dose, intervention time period, and the population of children and adolescents on which its effect were assessed (clinical or non-clinical group).

Previous work by the current authors highlighted the safety and efficacy of single extracts of *B.monnieri* in children and adolescents [5]. The primary outcome of the current review was to summarise the evidence for *B.monnieri*-dominant formulations in terms of cognitive and behavioural outcomes. Secondary outcomes included an examination of the safety and tolerability of *B.monnieri*-containing formulations and their viability as alternative treatments in clinical populations.

## 2. Materials and Methods

Pubmed, Scopus, Cochrane Library, DHARA, Google Scholar, and CINAHL were searched up to April 2017 for trials with child and adolescent populations examining the cognitive and behavioural effects of poly-herbal formulas containing *B.monnieri*. There were no restrictions in terms of study design. The following terms and truncations were searched: cognit*, executive function, neurocognit*, memory, intelligence, behaviour, and attention. These terms were searched against the following: *Bacopa monnieri (L.) Wettst.*, *Bacopa monniera,* or *brahmi*. The reference lists of any relevant papers were also examined for trials with a similar design. Websites promoting extracts containing *B.monnieri* meeting inclusion criteria of this review were also investigated. The following inclusion criteria were used:

Inclusion criteria:

1. Randomised, double-blind, controlled trial design;
2. Investigating an extract *B.monnieri* dominant extract;
3. Sample consisting of children or adolescents (aged 4–18);
4. Participants were not taking any other intervention during study period;
5. Sample size ≥20 (10 if a cross-over study);
6. Duration of intervention ≥1 month;
7. Have measurable outcomes on cognition or behavior;
8. Full paper in English.

### 2.1. Effect Size

For each study, Cohen’s *d* effect size calculations were performed on significant data and were reported in one of two ways [19]. The first utilised the significant differences between treatment groups (ES) at study end (e.g., treatment vs. placebo). The second calculated a treatment effect size (TES), in which only change scores were available (treatment group baseline vs. treatment group study end). Effect sizes were reported in all studies where the results were significant (small clinical effect = 0.2, medium = clinical effect 0.5, and large clinical effect = 0.8). Effect sizes were not calculated for non-significant results or when data was not appropriate to perform calculations (ES: N/A). To enable a comparison of the studies, the total amount of *B.monnieri* for each study, per participant, per day was calculated.

### 2.2. Behavioural Data

Behavioural outcomes were grouped into constructs based on the ADHD framework, as described and validated by the Diagnostic and Statistical Manual 5th edition (DSM-5) [20]. These behavioural domains comprise of symptoms that have been the subject of confirmatory factor analyses [21]. These symptoms include hyperactivity—described as having difficulty remaining seated, fidgeting with hands or feet, excessive running, or climbing or always being on the go; inattention—described as losing things, difficulty organizing work, being easily distracted, having difficulty sustaining attention, having difficulty following instructions, not listening, and not finishing tasks; impulsivity—described as difficulty waiting and interrupting, engaging in dangerous activities without considering consequences, blurting out answers, and acting before thinking [21]; peer relations—described as having no friends, losing friends, not making friends, not getting invited, and feeling inferior [22]. Confirmatory factor analysis of the DSM-IV ADHD rating scales has previously indicated increased variance in teacher- and parent-rated behaviours [23]. In order to address this variance, any teacher and parent reports of behaviour were assessed separately.

### 2.3. Cognitive Data

Any cognitive testing was assessed in terms of its true cognitive ability, as described by Carroll (1993) [24]. These are outlined in Pase et al. (2012) and include aspects of cognitive performance such as: reasoning—incorporating general, quantitative, syllogistic, and verbal reasoning, as well as induction; language behavior—incorporating vocabulary, spelling ability, phonetic coding, and verbal comprehension; memory—associative memory, free recall, visual memory, and memory span; visual perception—incorporating figural relations, closure speed, and perceptual speed; auditory perception—incorporating pitch discrimination; number facility—incorporating the ability to compute basic numerical operations; mental speed–processing speed and simple reaction time; and idea production—incorporating abilities in producing words, ideas, and figural creations such as originality and word fluency [25]. Symptoms of specific learning problems were described as difficulty with reading or writing, problems with math skills, difficulty remembering, problems paying attention, trouble following directions, poor coordination, difficulty with concepts related to time, and problems staying organized [26]. For brevity, only randomised controlled trials (RCTs) were included in the true cognitive ability and behavioural domain tables.

### 2.4. Methodological Quality Rating

Finally, the methodological quality of the research article was rated using a modified Jadad Scale [27]. This scale highlights key components of each study, which includes randomisation, blinding, and withdrawals. The modified version highlights exclusion criteria, intervention used, control used, and reported data [28].

Modified Jadad Scale:

1. Was the study described as randomised?
2. Was the randomisation protocol detailed and appropriate?
3. Was the study described as double-blind?
4. Was the blinding process detailed and appropriate?
5. Did the study have a control group?
6. Was the control detailed and appropriate?
7. Was there an adequate exclusion criteria?
8. Was the intervention used at a therapeutic dose?
9. Was there a description of withdrawals and dropouts?
10. Were the data clearly and adequately reported?

Yes = 1 point; No = 0 points; Total/10.

## 3. Results

**Figure 1:**
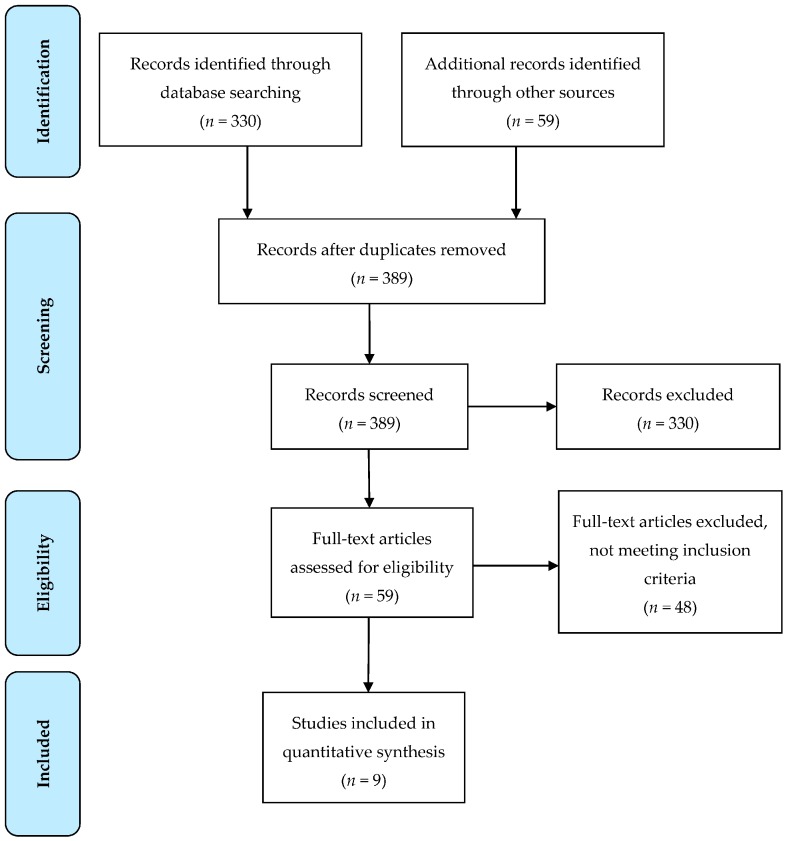
Flow chart of trial search for inclusion in review using PRISMA method.

Nine studies were identified as investigating poly-herbal formulas with a dominant *B.monineri* component in child and adolescent populations with cognitive and/or behavioural outcomes in a randomised controlled design [29,30,31,32,33,34,35,36,37] (see Figure 1 for a flow chart of the included study trial search). See Table 1 for a list of ingredients for each poly-herbal formula. One study included two distinct components; the initial research design was completed and then followed up with a secondary research design [31]. This was divided into its two distinct designs, with only one meeting inclusion criteria for this review [31].

The reviewed studies reported varying levels of efficacy for study outcomes including behaviour, learning, and cognition. Secondary outcomes included an examination of the safety and tolerability of *B.monineri*-containing formulations and their viability as alternative treatments in clinical populations. Any additional physiological improvements are included in Table 2. All poly-herbal studies contained *B.monineri* as the main component. All studies were randomised controlled trials. The average dropout rate was 9.89%. Dropouts or withdrawals were not reported in one study [30]. Table 2 contains a detailed description of each study and the effect sizes for each outcome measure. One study reported *overall* scores for cognitive outcomes [37], and another reported overall scores for behavioural outcomes [31]. As such, individual sub-test data for these studies could not be verified. These outcomes are reported here; however, any individual sub-tests derived from this data should be interpreted with caution.

In order to quantify the strength of a significant result, Cohen’s *d* effects sizes were calculated for each study that reported treatment group scores (mean and standard deviation) at baseline and at the final visit. A variation of the Cohen’s *d* effect size was calculated for those studies that reported significant change scores in *B.monnieri*-containing formulation treated groups only. Effect size calculations were conducted on six studies [29,31,34,35,36,37], four were between-group analyses (treatment versus placebo: [29,31,36,37], and two were treatment effects analyses (treatment baseline versus treatment final visit: [34,35]). To enable direct comparison of the studies, the authors of the current review (JDK) calculated the total amount of *B.monnieri* administered for each study, per participant, and per day (see Table 2); *B.monnieri* dosages ranged between 144 mg to 544 mg per day.

### 3.1. Cognition

Studies providing sufficient data for assessment within the Carroll cognitive abilities framework demonstrated a consistent positive effect for the visual perception domain (see Table 3) [29,31,34,35,36,37], with two studies reporting distinct sub-test improvements [35,36]. Three studies reported sub-test outcomes classified under the reasoning domain [31,34,37], of which only one reported distinct sub-test results (non-significant) [34]. Three studies included sub-tests investigating the language behaviour domain [29,34,37], with only one study demonstrating significant sub-test improvements following treatment [29]. Two studies investigated number facilities [34,37], with only one demonstrating significant sub-test effects [34]. Two studies investigated the mental speed domain [34,37], with one study reporting significant improvements [34]. In terms of memory, two studies included sub-tests within the free recall memory domain [34,37], with one demonstrating improvements [37]. Two studies included sub-tests within the associative memory domain [34,37], with two demonstrating improvements [37]. One study reported significant improvements in the auditory memory domain [3]. No studies collected data within the sub-tests for memory span, visual memory, and meaningful memory. As the data for two studies were not reported individually, the benefits to specific domains should be interpreted with caution [31,37].

### 3.2. Learning

Learning was added to the cognitive domains, as it explores a distinct element of child and adolescent development not commonly seen in adult cognitive research (see Table 3). Two studies included sub-tests that explored elements of learning [29,37], with only one demonstrating significant improvements following poly-herbal intervention [29].

### 3.3. Behaviour

Five studies investigated improvements in child and adolescent behaviour following treatment administration (see Table 3) [29,31,35,36,37]. Three of these studies reported significant improvements in behavioural sub-test results [29,35,36], with one reporting overall improvements [31] and another reporting no improvements [37]. Improvements in the symptom of impulsivity were noted in three studies [29,35,36], with one study showing no significant improvements [37]. Improvements in the attention domain were reported in three studies [29,35,36], with one study reporting no such improvements [37]. Symptoms of hyperactivity were investigated in four studies [29,31,35,37], with two reporting significant sub-test improvements [29,35]. One of the three studies investigating peer relations [29,31,37] reported improvements [29]. Only one study reported behavioural improvements based on the overall data of a behavioural outcome. Improvements were reported within the hyperactivity, peer relations, and aggression domains [31].

### 3.4. Quality Rating

The average quality rating for the nine studies was 6.78 (assessed by JK; see Table 4). The lowest rating was a 5 [30,31,32,34], and the highest was a 10 [36]. The most common error made by authors was a failure to provide a detailed description of the randomisation process [29,30,31,32,33,34,35].

Table 5 details the history and use of each ingredient from any poly-herbal extract included in this review.

## 4. Discussion

The current review lends some support for the use of poly-herbal interventions in child and adolescent clinical populations. The safety of each concoction requires significantly greater attention from the researchers, with 44% of the included studies not declaring any safety outcomes [29,31,33]. Four studies reported no treatment side effects experienced by the participants [29,31,33,34], with one reporting adverse events that were short-lived and mild in severity, with no difference between treatment groups [36]. The quality rating highlights design flaws that future research may need to focus on. The clear and concise description of the randomisation process, the blinding process, and the safety data collected is imperative to understanding how the study was conducted as well as how stringently the researchers adhered to protocols. Without these, the research and the treatment outcomes can be questioned as to their true validity.

Complementary and alternative medicines (CAM) are fast becoming viable standalone or adjunctive treatment preferences in place of pharmaceutical treatment for a range of developmental disorders including attention-deficit/hyperactivity disorder (ADHD) [5,28,38], mental retardation [31,39], autism spectrum disorders [40], and learning disorders [41,42]. This review investigated the cognitive and behavioural efficacy of the Ayurvedic medicine *B.monnieri* as the dominant ingredient within poly-herbal formulas in child and adolescent populations in both clinical and non-clinical settings. The Carroll methodology is an important framework for allowing the comparison of cognitive tests across studies. In the current review, this framework, along with the behavioural ADHD framework, highlighted the benefits of poly-herbal formulas on the distinct domains of visual perception, impulsivity, and attention. These domains play vital roles in children’s academic abilities, allowing them greater concentration and understanding as well as reduced error-making [43,44]. With the reporting of overall data, Memomet [37] demonstrated improvements in the reasoning, language behaviour, number facility, mental speed, free recall memory, associative memory, and auditory memory domains, while it was reported that Mentat [31] demonstrated overall benefits in reasoning, hyperactivity, peer relations, and aggression. However, extrapolating the distinct sub-test data would allow greater insight into each formulas’ benefits across these discrete cognitive and behavioural domains. For example, in an almost identical design to a study by Dixit et al. (1992), Upadhyay (2002) evaluated the formula Mentat in the same clinical population (learning disabled children) [34,41]. Where Dixit et al. reported significant overall outcomes, Upadhyay et al. extrapolated the sub-test results, identifying distinct improvements in the number facility and mental speed domains. Identifying these specific domains allows investigators to direct future research that may greatly benefit clinical and non-clinical populations.

A number of recent studies have reported the benefits of single-extracts of *B.monnieri* on human cognitive function in non-clinical populations [1,6,7,8,14]. Using this and similar supportive data, companies combine individual extracts, each with their own unique research-supported health benefits, to create poly-herbal remedies for various conditions. In the current review, the included studies demonstrated significant cognitive and behavioural improvements following supplementation with *B.monnieri*-dominant poly-herbal formulas. Improvements in hyperactivity and attention were consistent with previous research into *B.monnieri*, as well as improvements in language behaviour and memory free recall in both child and adolescent [5,45,46,47], as well as adult, [8,14,18,25,48] populations. The extent to which the additional herbal extracts may be working in synergism to aid these improvements is beyond the scope of this review. The evidence in this review highlights the need for increased design consistency, as well as stricter statistical analyses to ensure all future outcomes are comparable. One study included in this review utilised the Bannatyne categories for grouping WISC-R scores to better understand learning disorders in children and adolescents [34]. This method could be investigated further in future systematic reviews in order to understand the benefits of natural and poly-herbal interventions on the core symptoms of child and adolescent learning disorders.

In terms of the most common extracts included in the poly-herbal formulas, all of them included the Ayurvedic herb *Centella asiatica* (gotu kola) [29,30,31,32,33,34,35,36,37], renowned for its cognitive enhancing abilities [49] and cardiovascular health [50]. Three formulas included *Withania somnifera* (ashwagandha) [29,30,31,32,33,34,35,36] a herb known for its anti-anxiety and anti-stress effects [51]. Two formulas included *Convolvulus pluricaulis* (shankhpushpi) [35,37], an Ayurvedic herb also known for its cognitive enhancing abilities [52]. Two formulas included *Nardostachys jatamansi* (Jatamansi) [29,30,31,32,33,34,35], utilised for its calming effects [53], as well as its anticonvulsive [54] and antiepileptic [55] activities. Two formulas included *Embelia ribes* (false black pepper) [29,30,31,32,33,34,35], which has reported benefits in neuroprotection [56], as well as having antibiotic properties [57]. The different formulations included in this review make it difficult to directly compare each study; however, the crucial outcome this review offers is that a multitude of CAM treatments are being used to treat this vulnerable population that require more stringent and detailed study protocols.

The Ayurveda medicinal system employs a holistic approach to health utilising the synergistic properties of organic resources. Despite each of these distinct ingredients having demonstrated efficacy for multiple health conditions, they require greater scientific validation for their use as an individual CAM treatment, let alone as a part of a synergistic multi-herbal formulation. Strictly monitored clinical trials, such as the safety and tolerability study conducted by Chauhan and colleagues [58] on the poly-herbal formula Mentat, are essential to fully understand the safe use and limitations of any formula when treating clinical and non-clinical populations. Developmental disorders need structured, multi-dimensional forms of treatment for the best possible academic, social, and mental health outcomes for children and adolescents [20]. Furthermore, poly-herbal formulas highlight an area of research and development that requires significantly greater input from experts within the realm of alternative treatment so as to monitor, discuss, and direct future research programs such as those featured in this review.

The strength of this review is that a rigorous search was conducted and detailed analysis of each included study was undertaken. A weakness of this review is that only studies published in English were included. *B.monnieri* is native to India and, following 3000 years of its use in the Ayurvedic medicinal system, studies in languages other than English must exist. Table 5 details the efficacy of all of the included herbs on human health; however, it was not feasible to investigate the interactions or synergistic effects these herbs may have with one another. The large number of easily accessible combination formulas highlights the importance of understanding the research behind them, as well as their safe use in child and adolescent populations.

The current review indicates that poly-herbal formulas containing *Bacopa monnieri (L.) Wettst.* may alleviate behavioural symptoms and improve cognitive outcomes in children and adolescents with developmental disorders.

## Figures and Tables

**Table 1:** Ingredients within each included poly-herbal formula.

Formula	Ingredient	mg
Mentat	*Bacopa monnieri*	144
	*Centella asiatica*	70
	*Withania somnifera*	52
	*Evovulus alsinodes*	52
	*Nardostachys jatamansi*	52
	*Valeriana wallichii*	50
	*Embelia ribes*	50
	*Prunus amygdalus*	50
	*Tinospora cordifolia*	36
	*Terminalia chebula*	36
	*Emblica officinalis*	36
	*Oroxylum indicum*	32
	*Celastrus paniculatus*	32
	*Mucuna pruriens*	1.8
	*Elettaria cardamomum*	1.8
	*Terminalia arjuna*	1.8
	*Foeniculum vulgare*	1.8
	*Ipomoea digitata*	1.8
	*Orchis mascula*	1.8
	*Zingiber officinale*	1.4
	*Terminalia belirica Syn. T.bellirica*	1.4
	*Myristica fragrans*	1.4
	*Syzygium aromaticum*	1
Manas Niyamak Yoga Granule * (MN1 Granule)	*Bacopa monnieri*	N/A
	*Centella asiatica*	N/A
	*Convolvulus pluricaulis*	N/A
	*Nardostachys jatamansi*	N/A
	*Acorus calamus*	N/A
	*Withania somnifera*	N/A
	*Embelia ribes*	N/A
	*Glycyrrhiza glabra*	N/A
	*Plumbago zeylanica*	N/A
	*Piper longum*	N/A
Compound Herbal Preparation (CHP)	*Bacopa monnieri*	N/A
	*Paeoniae alba*	N/A
	*Withania somnifera*	N/A
	*Centella asiatica*	N/A
	*Spirulina platensis*	N/A
	*Melissa officinalis*	N/A
Memomet	*Bacopa monnieri*	125
	*Convolvulus pluricaulis*	100
	*Centella asiatica*	100

* Treatment also included Shirodhara: milk poured over the forehead of the participant from a height of 3.14 inches, oscillating left to right for 30–45 min/day. N/A—amounts not available; mg—milligrams.

**Table 2:** Poly-herbal formula studies in child and adolescent clinical and non-clinical populations.

Author	Intervention	*n*	Male (%)	Duration	Population	Safety	Dropouts (%)	Results (ES)
D’souza & Chavda (1991)	Mentat × 1–2 Tsp p/d (BM: 144–288 mg p/d) PL × 1–2 Tsp p/d	60 30 (B) 30 (P)	82	3-months	3–16 yrs Behavioural Problems	Not declared	0	YBI- Hyperactivity (ES: 6.55); Tractability (ES: 5.57); Habituation (ES: 5.20); Negative effects (ES: 4.31); Social (ES: 4.88); Academia (ES: 3.52); Impulsivity (ES: 5.10)
Patel & Pereira (1991)	Mentat × 2 Tsp p/d (BM: 288 mg p/d) PL x 2 Tsp p/d	40 20 (B) 20 (P)	N/A	3–7-months	2–7 yrs approx. Hyperkinesis	Nil side effects reported by participants	N/A	YBI-ES: N/A I.Q.-ES: N/A
Dave et al. (Study I) (1993)	Mentat × 2 Tsp 3/d (BM: 864 mg p/d) PL × 2 Tsp 3/d	19 10 (B) 9 (P)	N/A	3-months	1–18 yrs Mental retardation	Not declared	5	CBI-ES: 5.77
Quadri (1993)	Mentat × 2–4 Tabs p/d (BM: 272–544 mg p/d) PL × 2–4 Tabs p/d	50 30 (B) 20 (P)	70	20-months	4–12 yrs Mental retardation Behavioural issues	Nil side effects reported by participants ^a^	12	Behavioural Changes-ES: N/A
Kalra et al. (2002)	Mentat × 2 Tabs p/d (BM: 272 mg p/d) PL × 2 Tabs p/d	60 30 (B) 30 (P)	78	6-months	6–12 yrs ADHD Diagnosed	Not declared	17	CPRS-ES: N/A
Upadhyay et al. (2002)	Group N-PL Group P-Mentat Group S/F-PL Group R/G-Mentat (BM: unknown)	100 25 (P) 25 (B) 25 (P) 25 (B)	0	6-months	11–16 yrs Learning disability	Not declared ^b^	5	Group P-Coding (TES: 1.48); Sequential (TES: 1.77); Full scale I.Q. (TES: 0.94); Arithmetic (TES: 1.41); Digit Span (TES: 1.14). Group R/G-Coding (TES: 1.95); Sequential (TES: 2.45); Full Scale I.Q. (TES: 1.78); Performance I.Q. (TES: 1.40); Arithmetic (TES: 2.39); Digit Span (TES: 1.52).
Ojha et al. (2007)	A-MN1 × 200 mg/kg/d B-MN1 × 200 mg/kg/d + *Shirodhara* ^c^ C-PL × 2/d D-PL + *Shirodhara* (BM: unknown)	48 ^d^ 10 (B) 10 (B) 10 (B) 10 (P)	80 *	3-months	6–15 yrs ADHD Diagnosed	Nil side effects were reported by participants	17	ADHD Sx—ES: N/A CDA (TES: A—1.96; B—4.49; D—1.38) RT (TES: A—3.73; B—6.74; D—4.24).
Katz et al. (2010)	CHP × 3 mL 3/d PL × 3 mL 3/d (BM: unknown)	120 80 (B) 40 (P)	77 *	4-months	6–12 yrs ADHD Diagnosed	Safety well monitored. Few adverse events were reported ^e^	18	TOVA: Response time (ES: 0.70) Variability (ES: 1.02) Overall (ES: 1.11).
Dutta et al. (2012)	Memomet × 1 Tsp 2/d (BM: 250 mg p/d) PL × 1 Tblsp 2/d	86 56 (B) 30 (P)	86	4-months	6–12 yrs ADHD Diagnosed	Nil side effects were reported by participants	15	MISIC (ES: 0.90) CPRS (ES: 0.86) YBI (ES: N/A)

BM—*B.monnieri*; PL—placebo; MN1—Manas Niyamak Yoga Granule; CHP—Compound Herbal Preparation; mg—milligrams; mL—millilitres; mL/kg—millilitres per kilogram; Tsp—teaspoon; Tab—Tablet; Tabs—Tablets; p/d—per day; 2/d—twice a day; 3/d—three times a day; 4/d—four times a day; (B)—*B.monnieri*; (P)—placebo; N/A—not available. yrs—years; ADHD—Attention-Deficit/Hyperactivity Disorder; CPRS—Conners’ Parent Rating scale; I.Q. —intelligence quotient; MISIC—Malin’s Intelligence Scale for Indian Children; ES—Effect Size (Cohen’s *d* – treatment versus placebo); TES—treatment effect size (Cohen’s *d*—change from baseline); ES: N/A—Effect size not available; CBI—Children’s Behavioural Inventory; RT—Reaction Time; * Percentage is based on males who completed the study. Sex of dropouts was not reported, so percentage of male participants may be higher. ^a^ Four Children dropped out of Mentat group, and two dropped out of placebo group; ^b^ authors stated that no child showed any behaviour or speech abnormalities during the trial—it is not clear as to whether they are referring to possible side effects; ^c^ *Shirodhara* Treatment: milk poured over the forehead of the participant from a height of 3.14 inches, oscillating left to right for 30–45 min/day; ^d^ forty-eight participants were included in this trial; 8 dropped out. It is unclear which groups these 8 participants were assigned to prior to dropping out of the study; ^e^ all adverse effects were reported as “mild, transient and did not persist past the first two weeks of treatment”; there were no differences in reported adverse events between groups; control group suffered greater than 50% withdrawal from study.

**Table 3:** True cognitive ability and behavioural construct outcomes.

Cognitive Ability/Behaviour	D’souza (1991) (Mentat)	Dave (Study I) (1993) (Mentat)	Dutta (2012) (Memomet)	Katz (2010) (CHP)	Ojha (2007) (MN1 Granule)	Upadhyay (2002) (Mentat)
Reasoning		Conceptual Dysfunction ^a^	Picture Arrangement ^a^; Word Reasoning ^a^; Object Assembly ^a^			Picture arrangement; Object Assembly
Visual Perception			Block Design; Mazes	Response Time **	Reaction Time **	Picture Completion; Block Design; Mazes
Auditory Perception						
Language behaviour	Language *		Language; Comprehension ^a^; Vocabulary ^a^			Comprehension; Vocabulary
Number Facility			Arithmetic ^a^; Digit span ^a^			Arithmetic **; Digit span **
Mental Speed			Digit Symbol ^a^			Digit Symbol**
Memory						
Free recall Memory			Information ^a^			Information
Associative Memory			Similarities ^a^			Similarities
Memory Span						
Visual Memory						
Auditory Memory			Letter-Number Sequencing ^a^			
Meaningful Memory						
Learning	Academics *; Habituation *		YBS-Academic; YBS-Habituation			
Behaviour						
Hyperactivity	Hyperactivity *	Incongruous Behaviour^a^	CRS-Hyperactivity; YBS-Hyperactivity		DSM-Hyperactivity **	
Inhibition/Impulsivity	Impulsivity *; Tractability *		CRS-Impulsivity; YBS-Impulsivity; YBS-Tractability	Commission Errors **	DSM-Impulsivity *	
Attention	Attention *		YBS-Attention	Omission Errors **	DSM-Attention *; Coefficient of Division of Attention **	
Peer Relations	CD Socialized *	Incongruous Ideation^a^; Self-Depreciation ^a^	YBS-CD Socialized			
Aggression	CD Aggressive	Anger-Hostility ^a^	YBS-CD Aggressive			

* *p* < 0.05; ** *p* < 0.01; CD—Conduct Disorder; CRS—Conners Rating Scale; YCI—Yale Children’s Inventory; YBS—Yale’s Behavioural Scale; ^a^ Outcomes reported significant increase in total score in active treatment group; subtest scores not provided.

**Table 4:** Jadad Scale Quality Rating of Included Studies.

Modified Jadad Scale	DAVE	D’SOUZA	DUTTA	KALRA	KATZ	OJHA	PATEL	QUADRI	UPADHYAY
1. Was the study described as randomised?	1	1	1	1	1	1	1	1	1
2. Was the randomisation protocol detailed and appropriate?	0	0	1	0	1	0	0	0	0
3. Was the study described as double-blind?	1	1	1	1	1	1	1	1	1
4. Was the blinding process detailed and appropriate?	0	0	1	1	1	1	0	0	0
5. Did the study have a control group?	1	1	1	1	1	1	1	1	1
6. Was the control detailed and appropriate?	1	1	1	0	1	1	0	0	0
7. Was there an adequate exclusion criteria?	0	0	0	1	1	1	0	0	1
8. Was the intervention used at a therapeutic dose?	1	1	1	1	1	1	1	1	0
9. Was there a description of withdrawals and dropouts?	0	0	1	1	1	1	0	1	0
10. Were the data clearly and adequately reported?	0	1	0	1	1	1	1	0	1
Quality Rating	5	6	8	8	10	9	5	5	5

**Table 5:** History and description of each extract from poly-herbal formulas and their benefits on human health.

Poly-Herbal Formulas—All Extracts
**Centalla asiatica (*C.asiatica*)** is a perennial herbaceous creeper belonging to the family Umbellifere (Apiceae) and known in India as mandukparni as listed in the “Sushruta Samhita” an ancient Indian medical text. Clinical trials have investigated the effect of *C.asiatica* on vascular injury in adults with improved microcirculatory parameters in chronic venous hypertensive patients [59,60], capillary permeability [50], and oedema’s in people suffering venous hypertension [61]. More recent clinical trials found improvements in cognition and mood following large daily chronic doses (750 mg per day) in adults [49]. One clinical trial found improvements in general mental ability in mentally retarded children following six months administration [62].
**Withania somnifera (*W.somnifera*)**, or commonly known as Ashwagandha, is from the Solanaceae family and is widely used in the Ayurvedic medicinal system [63]. Research has investigated benefits on anxiety [64], cognition [65], psychomotor performance [66], and more recent studies on its effectiveness on age-related disorders, neuronal atrophy and synaptic loss [67]. A recent review demonstrated the safety of *W.somnifera* at 700 mg/day, 1000 mg/day, and 1250 mg/day in healthy human adults, with only one person withdrawing from the trial following adverse events at the lowest dose (increased appetite, libido, and hallucinogenic effects) [68].
**Evovulus alsinodes (*E.alsinodes*)**, also known as Dwarf Morning Glory or Shankhpushpi, is from the Convolvulaceae family and is deemed a “sacred flower” in Kerala, a south-west state on India’s Malabar coast [69]. Its traditional uses have been to treat symptoms of fever, cough and cold, venereal diseases, azoospermia, adenitis, depression, whereas specific to the Ayurvedic system it has been purportedly used as a “brain tonic” to aid neurodegenerative diseases, asthma and amnesia [70].
**Nardostachys jatamansi (*N.jatamansi*)**, from the Valerianceae family grows in the Himalayas of Nepal, China, and India. In Ayurveda it is classified as a hypno-sedative and has been used to treat insomnia, hysteria, and depressive illness [71]. In animal models, *N.jatamansi* has been found to not only improve learning and memory in mice, but reverse diazepam induced amnesia using a 200 mg/kg dose [72]. A study by Amin, Dixit, and Pathak (1961) investigated the effects of *N.jatamansi* (60 gm) on reaction times of medical students with significant sedative effects in the treatment group [73].
**Valeriana wallichii (*V.wallichii*)** is a rhizome herb and is another member of the Valerianaceae family. It is popularly known as Indian Valerian (a sometimes substitute for the European *V.officianalis*) [74]. Phytochemicals in Valerian have been used to treat mild to moderate insomnia [75] as well as gastrointestinal discomfort [76], as well as increased CNS activity in mice at 200, 400, and 600 mg/kg doses as well as increased relaxant activity at 800 mg/kg [77,78]. One human study highlighted the safety and efficacy of *V.wallichii* on stress related disorders at a dose of 1000 mg per day [79].
**Embelia ribes (*E.ribes*)** is from the species Myrsinaceae found throughout India, is also known as “False Black Pepper” or “Vidanga” from the Sanskrit “Vidang” [56]. It is commonly used for its antibiotic properties [57]. Recent work has investigated the significant antioxidant activities capabilities of *E.ribes* [56], and one study investigated the potentiality of *E.ribes* on acetylcholinesterase-inhibitory activity with significant results indicating possible cognitive benefits [80].
**Tinospora cordifolia (*T.cordifolia*)** from the Menispermaceae family, has been traditionally used in India’s Ayurvedic medicinal system [81] and Indonesia’s Jamu medicinal system [82]. It is well-known for its ability to statistically reduce symptoms of hay fever (allergic rhinitis) in adults [81] and has shown indications for the relief of constipation if taken regularly at high doses [83]. In a recent combination formula trial, *T.cordifolia* was found to be more beneficial for cognition when in combination with Bacopa and *E.alsinoides* than as a single extract in a rat model [84].
**Terminalia chebula (*T.chebula*)** from the *Terminalia* species is native to Asia, India, Nepal, China, Sri Lanka, and Vietnam [85]. It is a dried fruit that has exhibited various benefits including anticancer [86], antidiabetic [87], antimutagenic [88], antibacterial [89], antifungal [90], and antiviral properties [91]. A trial in Belgium school children (12–15 years old) found that a mouthrinse preparation of *T.chebula* substantially reduced salivary Streptococcus mutans compared to placebo, a clear demonstration of the herbs antibacterial properties. Further studies have validated the antioxidant and anti-inflammatory properties [92] of the herb.
**Emblica officinalis (*E.officinalis*)**, otherwise known as the Indian Gooseberry, is derived from a tree of the Phyllanthaceae species. Recent studies have demonstrated *E.officinalis* has excellent antioxidant activity in single extract form [93] and in polyherbal formulas [94], and has also demonstrated anti-inflammatory properties [95,96,97].
**Oroxylum indicum (*O.indicum*)** from the Bignoniaceae family is a large tree with trumpet-like flowers that bloom at night. Otherwise known as “Midnight Horror” (derived from the way its leaves wither and fall at its base resembling piles of broken bones), *O.indicum* is used for the treatment of diabetes by the Sikkim tribal people of India. Animal models have demonstrated *O.indicum*’s positive effects on antioxidant status, cholesterol and HDL levels and showed enhanced responses to insulin sensitivity [98].
**Celastrus paniculatus (*C.paniculatus*)** is a deciduous vine whose seeds are utilised for their fatty acid lipid content [99,100]. Indian researchers have found the oil derived from the plant is an effective acetylcholinesterase inhibitor and thereby is classed as a nootropic medication (memory enhancer) [101].
**Mucuna pruriens (*M.pruriens*)** produces a seed that contains L-DOPA, the precursor to dopamine neurotransmitters leading to investigations of its use in treating Parkinsons Disease [102,103,104,105,106]. The ethanolic extract of *M.pruriens* leaves has also demonstrated an antiepileptic and anticataleptic effect in animal models [107]. Unfortunately retrieving the seeds from the tree is quite tricky with each seed pod covered in spicules that contain serotonin causing severe itching, which is where its name feijões malucos the “mad beans” comes from.
**Elettaria cardamomum (*E.cardamomum*)** otherwise known as Green Cardamom contains a number of alkaloids, flavonoids, saponins, sterols, and tannins and has long been used to treat the symptoms of asthma [108]. A methanol prepared extract of *E.cardamomum* was shown to have significant antibacterial properties [109].
**Terminalia arjuna (*T.arjuna*)** was first used as an ancient Ayurveda treatment for the treatment of heart disease in 7th century by Indian physician Vāgbhata [110]. Its powdered tree bark contains glycosides, flavonoids, tannins and minerals [111]. Research around *T.arjuna* still focuses on its influence upon the cardiovascular system with newer trials investigating its phytochemical levels and their capabilities as an antibacterial remedy [112], an antioxidant, and anti-inflammatory agent [113].
**Foeniculum vulgare (*F.vulgare*)**, or Fennel, comes from the Apiaceae family. Known in Ayurveda as “Sanuf”, *F.vulgare* has previously been used to treat stomach pains, constipation, and intestinal tract issues [77,114], as well as acting as an analgesic [115], an anti-inflammatory [115,116], and having memory-enhancing effects [78].
**Ipomoea digitata (*I.digitata*)**, commonly referred to as Morning Glory or Aligator Yam, is one of 500 species of the Convolulaceae family. It is used most commonly as an antibacterial agent [117], however more recent therapeutic benefits include menorrhea, gastrointestinal disorders, and as a libido enhancer [118].
**Orchis mascula (*O.mascula*)** from the *Orchidaceae orchid* family, is a perennial herbaceous plant with small yet bright violet flowers. Modern uses for the plant are for the treatment of hypertension and dyslipidemia [119]. *O.mascula* has also demonstrated significant anticholinesterase enzyme inhibiting activity [120].
**Zingiber officinale (*Z.officinale*)**, the rhizome of the Ginger plant, has an extensive history in medicine as an anti-cancer intervention by reducing or slowing the growth of tumours in patients [121]. More recent studies have focused on the abilities of *Z.officinale* to reduce nausea and vomiting in various illnesses [122,123,124]. *Z.officinale* has also shown to be beneficial for reducing muscle pain following exercise [125], as well as having significant anxiolytic effects in animals models [126].
**Terminalia belirica (*T.bellirica*)**, or Bastard Myrobalan, has a history in Hinduism for being evil, with Hindus in the north of the country refusing to sit in its shade for fear of the tree being possessed by demons [127]. As a part of other herbal formulations, *T.bellirica* has shown to improve infected, inflamed or degenerating eye disorders [128], as well as having antiviral properties [129].
**Myristica fragrans (*M.fragrans*)**, or Nutmeg, is a spice native to Indonesia that has demonstrated anticholinesterase activity [130]. The spices antioxidant activity has been well documented [131,132,133,134], as well as having anti-inflammatory effects in the treatment of periodontitis [135]. In terms of memory, *M.fragrans* has shown significant memory improvements in mice models, which may or may not be attributed to its antioxidant, anti-inflammatory, or procholinergic activity [136].
**Syzygium aromaticum (*S.aromaticum*)**, or Clove, has significant antioxidant properties [137], attributed to its heightened levels of polyphenols [138]. Research has also found it has significant antimicrobial activity [139] as well as relieve neuropathic pain [140].
**Asparagus racemosus (*A.racemosus*)**, a root extract from the asparagus species, has potential as an anti-dandruff ingredient [141]. Further high performance thin layer chromatography (HPTLC) analysis has demonstrated that *A.racemosus* had high levels of flavonoids, has demonstrated immunomodulatory capabilities [142], and has shown protection against xanthine oxidase, an enzyme that generates reaction oxygen species [143].
**Acorus calamus (*A.calamus*)** is from the Acoraceae family and has been traditionally used as a fragrance [144]. Its uses are far reaching and include, but not limited to, nervous disorders, appetite loss, bronchitis, chest pain, colic, cramps, diarrhoea, digestive disorders, flatulence, gas, indigestion, rheumatism, sedative, cough, fever, bronchitis, inflammation, depression, tumors, haemorrhoids, skin diseases, numbness, general debility and vascular disorders [145].
**Xanthium strumarium (*X.strumarium*)**, known as Rough Cocklebur, is from the Asteraceae family. The fruit of the plant has been used extensively in China for rhinitis, tympanitis, urticarial, and arthritis [146]. Contrary to its benefits, the fruit and seeds of *X.strumarium* are quite toxic and need to be consumed with caution. Recent research has demonstrated X.strumarium also has potent anti-inflammatory activities [147].
**Convolvulous pluricaulis (*C.pluricaulis*)** is a herb that is more commonly known as Shankhapushpi. The herb, much like Bacopa, is known as a Medhya, a drug used to improve memory and intellect. It has demonstrated cognitive enhancing capabilities with respect to improved learning and memory enhancement [148]. When compared to *E.alsinoides*, C.pluricaulis displayed increased nootropic effects, but to a lesser extent that *E.alsinoides* [149].
**Glycyrrhiza glabra (*G.glabra*)**, or Liquorice, has significant anti-inflammatory and anti-excitotoxic properties, providing well documented neuroprotection through the inhibition of a high-mobility group box 1 protein (HMGB1) induction and release [150]. It has also been implicated in Chinese treatments for insomnia [151], as well as having immunomodulating, antispasmodic, and antiallergic properties that provide for significant improvements in cough suppression [152].
**Plumbago zeylanica (*P.zeylanica*)** is derived from the species Plumbago. The main research outcomes have found it to be a significant antimalarial compound [153], however there has been significant research into the capabilities of *P.zeylanica* in terms of skin diseases [154] and more significantly, in the treatment of cancer cell growth [155].
**Piper longum (*P.longum*)** is from the Piperaceae family and is otherwise known as Indian Long Pepper. Research has shown it has immunomodulatory and cytoprotective effects [156] as well as antidepressant effects [157] through the suppression of behavioural and biochemical induced changes following corticosterone injections in mouse models [158].
**Paeoniae Alba (*P.alba*)** has been traditionally used gout, osteoarthritis, fever, respiratory tract illnesses, and cough. As a part of another formula, *P.alba* demonstrated use as a potential Parkinsonian adjunct treatment in reducing the adverse effects of L-DOPA treatment [159].
**Spirulina platensis (*S.platensis*)** is a Cyanobacterium that was traditionally used by the Aztecs up until the 16th Century [160]. It has high levels of protein and has demonstrated improved exercise capacity in human trials [161]. However most recent clinical research conducted has investigated the effects of S.platensis on cancer cells in animal models [162,163,164].
**Mellissa officinalis (*M.officinalis*)** has been traditionally used as a mild sedative, an anxiolytic and a hypnotic medicine [165]. Recent research has examined its capabilities as a stress ameliorating intervention [166], memory-enhancing supplement [167], and as a gastrointestinal and anti-inflammatory treatment [168].
**Saussurea lappa (*S.lappa*)** has exhibited anticancer, anti-inflammatory, gastro protective activitives as well as anticonvulsant, anti-ulcerative, hepatoprotective antimicrobial and antiviral activity [169]. Alternate names for the plant include *costus* or *kuth root* and the activities it exhibits have been well established lending promise to future drug discovery.
